# Factores asociados a violencia de pareja en estudiantes de medicina: un estudio transversal

**DOI:** 10.15446/rsap.V25n3.107435

**Published:** 2023-05-01

**Authors:** Julio Cjuno, Janina Bazalar-Palacios, Edgar Bazán-Palomino, Marco Alvarado-Carbonel, Joel Figueroa-Quiñones, Roxana Aznarán-Torres

**Affiliations:** 1 JC: Lic. Psicología. M.Sc. Investigación Clínica. Escuela de Medicina, Universidad Cesar Vallejo. Piura, Perú. jccjunoc@ucvvirtual.edu.pe Universidad César Vallejo Escuela de Medicina Universidad Cesar Vallejo Piura Peru jccjunoc@ucvvirtual.edu.pe; 2 JB: Lic. Enfermería. M.Sc. Ciencias de Investigación Epidemiológica. Escuela de Medicina, Universidad Tecnológica del Perú. Lima, Perú. jbazalar@utp.edu.pe Universidad Tecnológica del Perú Escuela de Medicina Universidad Tecnológica del Perú Lima Peru jbazalar@utp.edu.pe; 3 EB: MD. Ph.D. Ciencias de la Salud. Escuela de Medicina, Universidad Cesar Vallejo. Piura, Perú. ebazanp@ucv.edu.pe Universidad César Vallejo Escuela de Medicina Universidad Cesar Vallejo Piura Peru ebazanp@ucv.edu.pe; 4 MA: MD. Familiar y Comunitario. Ph.D. Investigación Clínica y Traslacional. Escuela de Medicina, Universidad Cesar Vallejo. Piura, Perú. malvarado@ucv.edu.pe Universidad César Vallejo Escuela de Medicina Universidad Cesar Vallejo Piura Peru malvarado@ucv.edu.pe; 5 JF: Lic. Psicología. M.Sc. Investigación y docencia universitaria. Escuela de Psicología, Universidad Señor de Sipán. Chiclayo, Perú. joelfq.13@gmail.com Escuela de Psicología Universidad Señor de Sipán Chiclayo Perú; 6 RA: MD. Pediatra. M.Sc. Docencia Universitaria. Escuela de Medicina, Universidad Cesar Vallejo. Trujillo, Perú. roxanaznaran@gmail.com Universidad César Vallejo Escuela de Medicina Universidad Cesar Vallejo Trujillo Peru

**Keywords:** Violencia de pareja, violencia de género, estudiantes de medicina, universidad *(fuente: DeCS, BIREME)*, Intimate partner violence, gender violence, medical students, university *(source: MeSH, NLM)*

## Abstract

**Objetivo:**

Determinar los factores demográficos asociados a la violencia de pareja en estudiantes de medicina.

**Métodos:**

Se realizó un estudio transversal con una muestra de 588 estudiantes de medicina humana matriculadas en una universidad privada de Piura, Perú, durante el año académico 2021-2 (julio-diciembre), con una mediana de edad de 20 años, cuya mayoría (406) reportó una relación de pareja de menos de dos años (69,1%). Se utilizó la herramienta de detección de abuso de la mujer (WAST) para evaluar los indicadores de violencia de pareja. Para las variables numéricas y categóricas, se crearon análisis descriptivos. Asimismo, se desarrollaron regresiones lineales simples y múltiples para los modelos crudo y ajustado, usando Stata v. 15.0.

**Resultados:**

La violencia de pareja estuvo presente en dos de cada cinco estudiantes (40,1%; IC 95% 36,9-44,8%), y nueve de cada diez estudiantes tenían enamorado o novio (90,9%; IC 95% 88,4- 93,1%). En el modelo ajustado de regresión, las mujeres que tenían enamorado o novio reportaron 26% menos de prevalencia de violencia de pareja (RP 0,74; IC 95 % 0,55-1,00) en comparación con las que estaban casadas o convivían con su pareja.

**Conclusiones:**

La presencia de violencia aumenta con la edad y la duración de la relación, y los estudiantes casados o en pareja reportaron más violencia de pareja que los estudiantes en una relación de pareja o novio.

## Abstract (en)

**Objective:**

To determine the demographic factors associated with intimate partner violence in medical students.

**Methods:**

A cross-sectional study was carried out with a sample of 588 human medicine students enrolled at a private university in Piura, Peru, during the 2021-2 academic year (July-December), with a median age of 20 years, the majority of whom (406) reported a relationship of less than two years (69.1%). The Women's Abuse Screening Tool (wast) was used to assess indicators of intimate partner violence. For numerical and categorical variables, descriptive analyzes were created. Likewise, simple and multiple linear regressions were developed for the crude and adjusted models, using Stata v. 15.0.

**Results:**

Intimate partner violence was present in two out of five students (40.1%; 95% CI 36.9-44.8%), and nine out of ten students had a lover or boyfriend (90.9%; 95% CI % 88.4- 93.1%). In the adjusted regression model, women who had a lover or boyfriend reported 26% less prevalence of intimate partner violence (PR 0.74; 95% CI 0.55-1.00) compared to those who were married or cohabiting. with your partner.

**Conclusions:**

The presence of violence increases with age and the duration of the relationship, and students who are married or in a relationship reported more partner violence than students in a relationship with a partner or boyfriend.

La violencia de pareja íntima (VPI) ha sido identificada como un problema importante de salud pública y de los derechos humanos en todo el mundo 1. De acuerdo a la Organización de las Naciones Unidas (ONU) 2, 243 millones de mujeres y niñas han sido víctimas de violencia física y sexual por parte de personas cercanas a ellas, como su pareja o familiares, en el último año; la mayoría de los casos ocurre dentro del hogar. En México, 37,5% de los estudiantes universitarios reportaron ser víctimas de violencia de pareja, siendo los más afectados los que mantienen una relación de noviazgo y relaciones sexuales, con cifras más altas en los que están casados o viven en unión libre 3. En Chile, por su parte, se destacó que la prevalencia de la violencia psicológica basada en críticas, insultos, control y desconfianza afecta a más del 50% de la muestra estudiada; más de un tercio de este grupo declara una frecuencia de tres o más episodios. La violencia física, presente en una cuarta parte de la muestra, se manifiesta principalmente con pellizcos y empujones 4.

En Perú, la violencia no fue aislada, ya que se reportaron 18 439 casos de violencia contra la mujer y miembros del grupo familiar en un semestre de 2020, siendo 15 924 los casos (86%) relacionados con violencia contra la mujer. Además, se han denunciado diversos casos de violencia física (8 418 casos), violencia psicológica (7 277 casos), violencia sexual (2 537 casos) y violencia patrimonial (51 casos) 5. Otro estudio realizado entre estudiantes universitarios en Lima, Perú, descubrió una relación altamente significativa entre la violencia sutil y los celos, con un 75,6% de los participantes que admiten haber usado violencia sutil con sus parejas en algún momento de sus vidas 6.

Un historial de victimización por VPI física o violencia sexual entre adolescentes masculinos y femeninos o adultos emergentes se ha relacionado con conductas sexuales de riesgo, conductas violentas, mala salud mental, uso de sustancias, otras conductas de riesgo para la salud, mala salud y bajo rendimiento académico, según varios estudios 1,3,7. El ser testigo de violencia paterno-materna, tener un nivel educativo superior al de la mujer 8 y la resolución de conflictos se han relacionado con la violencia de pareja en la población joven 9.

La violencia de pareja es un problema relevante por el impacto en la salud física y mental 10, como también por el riesgo de que se transforme en un modelo estable, con serias consecuencias tanto en la vida familiar como en la vida conyugal. Considerando la alta prevalencia a escala mundial y la escasa información a escala local en la población objetivo, el objetivo del presente estudio fue determinar los factores demográficos asociados a la violencia de pareja en estudiantes universitarias peruanas.

## MÉTODOS

### Diseño de investigación

El presente estudio es una investigación descriptiva de corte transversal 11. Se incluyeron 588 mujeres estudiantes de medicina humana matriculadas en una universidad privada de la ciudad de Piura, ubicada en norte del Perú, incluir: en el semestre académico 2021-2 (comprende desde el mes de julio a diciembre), provenientes en su mayoría de la clase económica media. A través de un muestro no probabilístico por conveniencia, se incluyeron estudiantes mayores de 18 años que autorreportaron vivir bajo el mismo techo que su pareja o tener pareja como novio o novia y que además aceptaron participar en el estudio voluntariamente. Aquellas que no respondieron todas las preguntas del cuestionario en línea fueron excluidas.

### Variables e instrumentos

Se utilizó la herramienta de detección de abuso de la mujer (WAST) para evaluar la violencia de pareja íntima 12. Dicho instrumento originalmente contaba con siete ítems, pero se propone un ítem adicional que fue tenido en cuenta en la versión en español de México 13, con lo cual se llega ocho ítems, para determinar el grado de tensión y la dificultad existente en la relación de pareja, así como la presencia de episodios violentos, tanto físicos como sexuales y emocionales, que se pueden responder en una escala tipo Likert del 1 al 3, siendo 1 la opción de menor intensidad o frecuencia 14. Este instrumento fue adaptado al contexto peruano utilizando el criterio de tres jueces expertos en casos de violencia, quienes lo aprobaron con una ponderación de la V de Aiken de 0,89-1,00 y una confiabilidad óptima de 0,86. En cuanto a sus evidencias de validez de estructura interna, ha mostrado adecuados ajustes como GFI=0,997, CFI=0,994, TLI=0,992, SRMR=0,049 y RMSEA=0,058.

Adicionalmente, se consideraron las variables: edad (autorreporte en años), lugar de residencia (asentamiento humano, pueblo joven, urbanización, rural), ingreso económico familiar mensual del último mes (autorreporte en soles), tipo de familia parental de procedencia (nuclear, extensa, monoparental, reconstituida), religión (católica, evangélica, testigo de Jehová, mormón, adventista), tiempo de convivencia (autorreporte en meses), tiempo de relación de pareja o enamoramiento/noviazgo (autorreporte en meses), su padre pegó físicamente a su madre (sí, no), o su padre insultaba a su madre (sí, no).

### Procedimientos

Para llevar a cabo este trabajo, obtuvimos permiso de las autoridades de la facultad de Medicina de una universidad en Piura para recopilar datos y usar el nombre en nuestra investigación. Luego se coordinaron los horarios y las fechas de recolección de datos. La encuesta se entregó digital-mente a través de un formulario de Google (https://forms.gle/Lc83w6YM7ZTjdWSu5). Este documento presentaba primero el consentimiento informado, en el que se describía el propósito del estudio y los beneficios para los participantes. Las preguntas fueron presentadas en la ficha de recolección de datos una vez aceptada su participación.

### Análisis de datos

Las características de la población de estudio se resumieron utilizando medidas de frecuencia y porcentaje para resumir las variables categóricas y las medidas de tendencia central organizadas y dispersas para las variables continuas.

La violencia de pareja se trató como una variable binaria (>10 puntos) y se resumió en porcentajes. Ajustamos un modelo de regresión de Poisson con varianza robusta para determinar la violencia de pareja no ajustada, como se recomienda para estudios transversales con una prevalencia de un resultado binario del 10% 15. Con modelos de Poisson similares, también estimamos razones de prevalencia (RP) ajustadas por las covariables. En el modelo ajustado se incluyeron covariables ocupacionales que obtuvieron una p=0,10 en el modelo anterior no ajustado (edad, estado civil con novio o novio, relación menor a dos años, educación menor a tres años e ingreso familiar).

Calculamos intervalos de confianza del 95% y consideramos valores de p de 0,05 como significativos. El análisis estadístico se realizó con Stata 16.1 para Windows (Stata Corporation, College Station, Texas).

### Aspectos éticos

El presente estudio fue aprobado por el Comité de Ética de la Universidad Cesar Vallejo con Informe N.° 022-CE-FCS-UCV-21. Asimismo, respetó todos los principios éticos de investigación en humanos de la Declaración de Helsinki, tales como la autonomía, la confidencialidad y la justicia 16.

## RESULTADOS

### Características de los participantes

Se encuestó a 588 estudiantes del sexo femenino, con una mediana de edad de 20 años (RIQ = 5,0). Una cuarta parte cursaba el primero, tercero y quinto año de estudios durante la aplicación de la encuesta. Nueve de cada diez tenían novio o novio (90,9%; IC 95% 88,4-93,1%), y alrededor del 69% llevaba menos de dos años en una relación (IC 95% 65,2- 72,7%) (Tabla 1).

**Tabla 1:** Características de los participantes del estudio

Características		n = 588
Edad, mediana ± IQR		20,0 ± 5,0
Estado civil		
	Conviviente o casada	53 (9,0)
	Novia o enamorada	535 (90,1)
Tiempo de relación		
	Más de dos años	182 (30,9)
	Menos de dos años	406 (69,1)
Año de estudios universitarios		
	Primero	147 (25,0)
	Segundo	78 (13,3)
	Tercero	149 (25,3)
	Cuarto	61 (10,4)
	Quinto	153 (26,0)
Ingreso económico familiar, mediana ± IQR		1850,0 ± 2000,0
Tipo de familia	Nuclear	381 (64,8)
	Extensa	22 (3,7)
	Reconstituida	47 (8,0)
	Monoparental	138 (23,5)
Lugar de	Urbana	448 (76,2)
residencia	Rural	140 (23,8)
Religión	Evangélica	122 (20,8)
	Católica	466 (79,2)
Woman Abuse Creening Tool		
	Ausencia	348 (59,2)
	Presencia	240 (40,8)

Abreviaciones: IQR, rango intercuartílico.

Tres de cada cinco mujeres pertenecen a familias nucleares (64,8%; IC 95% 60,1-68,5%), y en promedio tienen un ingreso familiar de 1 850 soles (RIQ = 2 000). Finalmente, dos de cada cinco son víctimas de violencia de pareja (40,1%; IC 95% 36,9-44,8%) (Tabla 1).

### Las mujeres tienen el riesgo de sufrir violencia de pareja

En el modelo no ajustado detectamos cinco factores asociados con la violencia de pareja. La violencia de pareja de la mujer se asoció con la edad (RP 1,03; IC 95% 1,01-1,05), el estado civil con novio o novio (RP 0,60; IC 95% 0,48-0,75), relación menor de dos años (RP 0,75; IC 95% 0,61-0,91), menos tres años de educación (RP 0,80; IC 95% 0,66-0,97), e ingreso familiar (RP 1,00; IC 95% 1,00-1,00) (Tabla 2).

**Tabla 2:** Factores de riesgo asociados a la violencia de pareja

Variables	Modelo crudo		Modelo ajustado^*^	
	PR (IC 95%)	p	PR (IC 95%)	p
Edad	1,03 (1,01 - 1,05)	>0,001	1,01 (0,99 - 1,03)	0,305
Estado civil				
Casada o conviviente	Ref.		Ref.	
Enamorada o novia	0,60 (0,48 - 0,75)	>0,001	0,74 (0,55 - 1,00)	0,048
Tiempo de relación				
Más de dos años	Ref.		Ref.	
Menos de dos años	0,75 (0,61 - 0,91)	0,003	0,86 (0,69 -1,06)	0,176
Años de estudio				
>3 años	Ref.		Ref.	
<3 años	0,80 (0,66 - 0,97)	0,025	0,90 (0,73 - 1,10)	0,313
Ingreso familiar	1,00 (1,00 - 1,00	0,035	1,00 (1,00 - 1,00	0,142
Tipos de familia				
Nuclear	Ref.		-	-
No nuclear	1,03 (0,83 - 1,27)	0,787	-	-
Religión				
Evangélica	Ref.		-	-
Católica	0,92 (0,73 - 1,16)	0,501	-	-

Abreviaciones: PR: prevalence ratio; IC: intervalo de confianza.

^*^ Ajustado por variables que obtuvieron p<0,10 en el modelo no ajustado.

En el modelo ajustado, el estado civil con novio o novio tiene un 26% menos de prevalencia de violencia de pareja en mujeres (RP 0,74; IC 95%: 0,55-1,00) en comparación con cohabitantes o casadas.

## DISCUSIÓN

En este estudio determinamos los factores demográficos asociados a la violencia de pareja en mujeres universitarias peruanas. Encontramos que dos de cada cinco mujeres universitarias informaron haber sido víctimas de violencia íntima de pareja. Las mujeres universitarias que mantienen una relación de noviazgo y menor de dos años de relación se asociaron con la violencia íntima de pareja en mujeres universitarias.

Dos de cada cinco mujeres universitarias reportaron violencia de pareja íntima. Nuestros hallazgos fueron consistentes con un estudio realizado en un programa universitario de ciencias de la salud en España, que encontró que más de la mitad (66%) de los estudiantes habían experimentado algún tipo de violencia de pareja 17. Además, un estudio de estudiantes de medicina en Cuba encontró que el 62% percibía conductas violentas durante el noviazgo 7. Múltiples factores podrían explicar la presencia de violencia; por ejemplo, la pandemia de COVID-19 y el confinamiento han prolongado el periodo de convivencia de las parejas y limitado su interacción social, lo que puede haber exacerbado el problema de la violencia. Otro factor se presenta cuando la capacidad de la pareja para resolver problemas y comunicarse efectivamente es insuficiente, o cuando existen características personales y antecedentes violentos 18,19.

Descubrimos que la prevalencia de violencia de pareja entre estudiantes universitarios aumenta en un 10% por cada año de edad. Según un estudio de revisión, la incidencia de violencia en el noviazgo es un 10% menor en parejas jóvenes que en mujeres mayores 20. La edad como factor asociado a la violencia puede deberse a que a mayor edad también implica una relación de pareja más prolongada, y en esta etapa se produce la perpetración por parte de la pareja de conductas violentas, como control posesivo, manipulación, celos exagerados, amenazas o maltrato. Lo anterior puede normalizarse, lo que se acentúa con el paso del tiempo 21,22.

Descubrimos que las mujeres universitarias que vivían en pareja o casadas sufrían más violencia de pareja. Un estudio en México descubrió que las parejas casadas o que cohabitan experimentaron más violencia 23. De hecho, un estudio de revisión encontró que la prevalencia de violencia de pareja íntima en mujeres casadas era superior al 50% 24. Es probable que el comportamiento violento sea menos común en mujeres que solo están en una relación de noviazgo, a diferencia de las parejas que cohabitan, las cuales enfrentan una variedad de situaciones que podrían generar un conflicto, como dificultades económicas, poca tolerancia con la pareja, comunicación ineficaz, y otros factores que explican la mayor prevalencia de violencia 25.

Las mujeres con un tiempo de relación menor a dos años reportaron menos violencia, en comparación con aquellas que tenían más tiempo de relación. Este resultado coincide con un estudio con mujeres de Etiopía, el cual halló que las mujeres con una duración de relación mayor a seis años reportaban cuatro veces más violencia 26. Asimismo, otro estudio con universitarias chilenas reveló que aquellas parejas que tuvieron una relación larga estuvieron expuestas a una mayor violencia 27. Algunos estudios sugieren que el desarrollo de la violencia en la pareja suele ser progresivo, si no se detiene. Por ejemplo, en una pareja reciente pueden presentarse conductas violentas que pasan inadvertidas, sin embargo, estas pueden aumentar su frecuencia e intensidad y convertirse en maltrato recurrente 28. Nuestros resultados revelan que las estudiantes universitarias vienen reportando prevalencias elevadas de violencia de pareja y aquello es mayor conforme tienen más tiempo de relación de pareja. Nuestros resultados podrían ser un sustento para promover políticas de promoción de las parejas saludables libres de violencia, como programas complementarios para estudiantes que vienen entablando relaciones sentimentales en universidades peruanas.

### Fortalezas y limitaciones

Este es el primer estudio en el norte del Perú que se ha centrado en evaluar los factores asociados a la violencia en una muestra amplia (n=588) de mujeres estudiantes de la carrera de medicina. Además, los instrumentos utilizados garantizan validez y confiabilidad en los resultados encontrados. Sin embargo, es preciso mencionar, en cuanto a las limitaciones de los estudios, que se utilizó un muestreo no probabilístico intencional, lo que limita la generalización de los resultados hallados; además, la violencia puede ser explicada por distintos factores que no fueron tomados en cuenta en este estudio y deben considerarse para entender este fenómeno.

La presencia de violencia aumenta con la edad y el tiempo de relación, y los universitarios casados o convivientes reportaron más violencia de pareja. Estudios posteriores podrían tener en cuenta muestras de otras carreras universitarias, así como otros factores como el apego, la autoestima y las relaciones familiares que pueden influir en la violencia de pareja ♦

## References

[1] Stark L, Seff I, Hoover A, Gordon R, Ligiero D, Massetti G (2019). Sex and age effects in past-year experiences of violence amongst adolescents in five countries. Plos One.

[2] Organización de las Naciones Unidas (2021). La pandemia en la sombra: violencia contra las mujeres durante el confinamiento.

[3] Eréndira TCM, Gómez JMC, Mendoza LIB, Leticia RÁ (2016). Factores asociados a la violencia de pareja, en universitarias del área de la salud del estado de Guerrero, México. Salud Probl.

[4] Larrañaga MBV, Figueroa AMP (2011). Violencia de pareja en estudiantes universitarios del sur de Chile. Univ Psychol.

[5] Ministerio de la Mujer y Poblaciones Vulnerables (MIMP) (2020). Estadísticas del MIMP.

[6] Tasayco CP, Sánchez DT, Rengifo CA (2019). Violencia sutil y celos en una relación de pareja en estudiantes de una universidad pública de Lima Este. Rev Científica Cienc Salud.

[7] Ramos Rangel Y, López Angulo LM, Suz Pompa M, García Ramos D, Ramos Rangel Y, López Angulo LM (2021). Caracterización de la violencia en el noviazgo en estudiantes de Medicina de la provincia de Cienfuegos. Rev Méd Electrón.

[8] Blitchtein-Winicki D, Reyes-Solari E (2012). Factores asociados a violencia física reciente de pareja hacia la mujer en el Perú, 2004-2007. Rev Peru Med Exp Salud Publica.

[9] Iskandar L, Braun KL, Katz AR (2015). Testing the woman abuse screening tool to identify intimate partner violence in Indonesia. J Interpers Violence.

[10] Organización Mundial de la Salud (2021). Violencia contra la mujer.

[11] Gordis L (2019). Epidemiología.

[12] Cjuno J, Rengifo CA, Palomino EB, Ramírez RG, Bardales RP, Carbonel MA (2022). Adaptation and validation of the woman abuse screening tool (WAST) in Peruvian university students. Arch Venez Farmacol Ter.

[13] Fogarty CT, Brown JB (2002). Screening for abuse in Spanish-speaking women. J Am Board Fam Pract.

[14] Binfa L, Cancino V, Ugarte I, Mella M, Cavada G, Binfa L (2018). Adaptación del instrumento WAST para la detección de violencia doméstica en centros de salud. Rev Médica Chile.

[15] Barros A, Hirakata V (2003). Alternatives for logistic regression in cross-sectional studies: an empirical comparison of models that directly estimate the prevalence ratio. BMC Med Res Methodol.

[16] Asociación Médica Mundial (2018). Declaración de Helsinki.

[17] Llano-Suárez A, Lana A, Gasch-Gallén Á, Fernández-Feito A (2021). Gender roles and intimate partner violence among female university students in Spain: A cross-sectional study. Plos One.

[18] Wang L (2016). Factors influencing attitude toward intimate partner violence. Aggress Violent Behav.

[19] Lyons M, Brewer G (2022). Experiences of intimate partner violence du-ring lockdown and the COVID-19 pandemic. J Fam Violence.

[20] Jennings WG, Okeem C, Piquero AR, Sellers CS, Theobald D, Farrington DP (2017). Dating and intimate partner violence among young persons ages 15-30: Evidence from a systematic review. Aggress Violent Behav.

[21] Kim J, Gray KA (2008). Leave or stay? Battered women's decision after intimate partner violence. J Interpers Violence.

[22] Mannell J, Willan S, Shahmanesh M, Seeley J, Sherr L, Gibbs A (2019). Why interventions to prevent intimate partner violence and HIV have failed young women in southern Africa. J Int AIDS Soc.

[23] Rubia JM de la, Rosales FL (2013). Violencia de pareja en personas que viven o no con su pareja y en ambos sexos. Psicogente.

[24] Elghossain T, Bott S, Akik C, Obermeyer CM (2019). Prevalence of intimate partner violence against women in the Arab world: a systematic review. BMC Int Health Hum Rights.

[25] Giordano PC, Soto DA, Manning WD, Longmore MA (2010). The characteristics of romantic relationships associated with teen dating violence. Soc Sci Res.

[26] Feseha G, G/mariam A, Gerbaba M (2012). Intimate partner physical violence among women in Shimelba refugee camp, northern Ethiopia. BMC Public Health.

[27] Vizcarra Larrañaga MB, Póo Figueroa AM (2011). Violencia de pareja en estudiantes universitarios del sur de Chile. Univ Psychol.

[28] Shitu S, Yeshaneh A, Abebe H (2021). Intimate partner violence and associated factors among reproductive age women during COVID-19 pandemic in Southern Ethiopia, 2020. Reprod Health.

